# Progress in General Surgery

**Published:** 1901-06-15

**Authors:** 


### PROGRESS IN GENERAL SURGERY.

**Fractures and Dislocations.**—Mr. D'Arcy Power,[1] in his recent paper "On Some Disappointments of Surgery," says that perhaps the most disastrous, but at the same time some of the best-known, disappointments occur in connection with injuries to bones. For example, there is the trouble after a Pott's fracture which has been well set shortly after the accident; the slight eversion and accompanying flatness of the foot are always causes of annoyance to the patient. But even when there has been a seemingly good result, and time has been allowed to obtain firm union, the original deformity may recur if the patient be unusually heavy, or if for any reason the amount of repair has been miscalculated. The difficulty is to restrain the patient from getting about and attending to his business at the earliest possible opportunity. It is far better for him, and in the end shorter, to wait until the union is thoroughly sound. Colles's fracture is fruitful in disappointments, and especially when it occurs in old people. Union takes place readily enough, but the connective tissue surrounding the joint becomes so infiltrated with the products of chronic inflammation as to leave a stiff and useless wrist for many months. It is of the utmost importance, therefore, to shampoo the injured part and to begin passive movement on the fourth day. Non-union is more than disappointing after the fracture of a long bone, for, in children at any rate, it leads to the most disastrous results, since it is often necessary to amputate the limb. In discussing the pathology of fractures Dr. Lewis Schooler[2] said that each case should be treated upon its merits, and the surgeon should have a thorough knowledge of all methods and appliances, as well as a knowledge of mechanical principles. The difference in the length and tension of muscles in the flexed and extended position of limbs was easily ascertained, and might be studied by anyone. The difficulty with text-books was a too strict adherence to former customs. Experience showed the difficulties and inability of these methods to meet the requirements in many cases. Mr. W. A. Lane[3] gives some instances of operative procedures for simple fractures which have been unscientifically treated. He commenced to operate on simple fractures in 1893, before the radiograph was known, and his views on this subject are well-known. When he considered the unfortunate physical condition of the patients whom he had had under his care, and the misery, distress, and financial loss they and those dependent on them have experienced through the gross inefficiency of the so-called science of which they are the victims, it seemed little short of ridiculous to read the statements of surgeons that such mechanical disability is a rare sequence of fracture, and that it can usually be obviated by the use of massage and passive movements at an early date. Massage and passive movements serve to diminish the disability and pain; but to suggest such measures alone hinted to a want of perception of the mechanics of the skeleton. According to Scudder[4] the open treatment of fractures should be undertaken only after careful consideration of the health of the patient, and under the most rigid antiseptic and aseptic conditions possible. It should be carried out only by a skilled and competent surgeon, and should not be done in the aged. It is an operation for young adults, particularly for the labouring man. Early massage and passive and active motion after four weeks are desirable in fracture after operation. It is impossible to determine which method of those in use to-day will prove effective until the fracture is exposed to view. But that method is the best which preserves the normal line of the shafts of the bones. In his recent reprint, in book form, "On the Use of Massage and Early Passive Movements in Recent Fractures and other Common Surgical Injuries," Mr. W. H. Bennett[5] says that the tendency of late has been to exaggerate the degree of disability and diminution in wage-earning capacity following upon simple fractures. Although no pains should be spared in obtaining perfect position of the fracture ends, moderate displacement, provided that it is not *rotatory*, is not necessarily followed by any disability, if care be taken by the use of early movements to prevent any matting of the parts around the fracture. A writer in the *Medical Record*[6] believes that in bone it is very rarely necessary to use a metallic suture, for it is never certain that there will not be some sort of irritation, which will make a secondary operation of greater or less extent necessary in order to remove the offending unabsorbed suture. The present supply of absorbable suture material is by no means satisfactory in all particulars, but it is improving continually. The ideal suture is that made of a strong, sterile, absorbable material which does not slip when tied; and these requirements are so nearly met in the best specimens of prepared catgut that it is in this direction that we should seek improvement which may lead to perfection. F. J. Cotton[7] calls attention to the comparatively unrecognised condition of sub-periosteal fracture. Although ordinary green-stick fractures are sub-periosteal, yet they present some deformity; whereas in sub-periosteal fractures there is a clean-cut crack or transverse fracture and no deformity. Crepitus is absent. These fractures are common in children, but before the introduction of skiagraphy into surgery they escaped notice. The writer describes several cases of this injury, in all of which a skiagram revealed a distinct crack across the bone not far from the epiphysial line. According to Von Bergmann[8] the increased knowledge of fractures has led to progress in two directions: the one, the treatment of certain simple fractures by open operation; the other, the diagnosis of the exact character of the fracture and the extension of pathological knowledge regarding osseous lesions which the Roentgen method has secured. The exploration of fractures by this method shows these obstacles to union: 1. The inequality of the two fragments; the upper fragment is much larger than the lower, and cannot be held in contact except by a suture. 2. The comminution of the bone into multiple fragments; the small pieces or splinters fall between the larger and prevent co-optation and union. 3. One of the fragments is displaced with rotation in such a manner that the fracture-surface of the other fragment being in contact with its entire surface, the rough surfaces cannot come into apposition and unite. Fracture of the lower end of the radius should, he thinks, probably be placed in the operative category with those of the patella, and undoubtedly better results would be obtained in many instances. Fractures of the tarsal and metatarsal bones can now be demonstrated, which were for a long time almost unknown, the symptoms being often mistaken for inflammatory conditions or contusions of the feet. A. B. Blacker[9] tells us that skiagraphy in dislocations is much less useful than in fractures, although in some cases where such an injury has been overlooked it has been found serviceable. But up to the present time it has given no indication as to the most frequent cause of irreducibility, which lies especially in the soft parts. In old dislocations it shows the newly-formed bone production which opposes reduction, and it verifies the presence of false joints. The further development and application of the X-rays to medicine and surgery is a study which J. H. Morgan[10] would like to see appropriated by members of the profession. He believes that it is still in its infancy, and that there is a great future before it. But in order to obtain the full advantages of its resources the study must be pursued by men having a thorough medical training, and must not be allowed by them to fall into the hands of mechanicians, lest its progress as a scientific aid to diagnosis be checked. He heartily commends it as a new form of specialty to those who have time and opportunity to pursue this entrancing study. E. W. H. Shenton[11] says that when the rays get into disgrace, it is generally because they have been relied upon in opposition to, or unassisted by, other more useful methods of diagnosis. It is not likely that in the X-ray instrument we shall have a machine that will replace all other ways of examination, nor yet one that will replace the medical man himself, as some have feared. The certainty that is conveyed to the mind of the practitioner by the confirmation of his own previously formed opinion is, perhaps, one of the most valuable points in skiagraphy. As regards fracture at the elbow-joint in children after a fall upon the extended arm, Shenton finds that in 50 per cent. of all children's elbows there is a fracture of the lower end of the humerus, with the general direction of downwards and forwards. Very often it is associated with a long splinter of bone attached to the lower fragment, but belonging originally to the back of the shaft of the humerus. An excellent skiagraphic study of the normal epiphyses of the long bones of the extremities at the thirteenth year has been produced by Dr. E. R. Corson,[12] and ought to afford a good basis for the clinical study of injuries of the extremities at this age. The descriptions of the development of the epiphyses, he says, have been largely taken from Mr. John Poland's researches and recent works on "Traumatic Separation of the Epiphyses" and "Skiagraphic Atlas of the Bones of the Wrist and Hand." The article is essential to surgeons who take an interest in difficult injuries of the bones at this age. After discussing the symptoms ordinarily met with in sprains Mr. A. H. Tubby[13] alludes to the practice of treating sprained joints by the method of mobility from the first. He thinks that it is one which can only be employed in very slight cases, and even then with some misgiving, for in certain patients the symptoms, instead of rapidly clearing away, become more pronounced, and permanent thickening and disability of the joint remains.

[1] Lancet, December 22, 1900, p. 1789. [2] New York Med. Rec., January 12, 1901, p. 74. [3] Edin. Med. Jour., January 1901, p. 45. [4] Amer. Jour. of Med. Sciences, January 1901, p. 102. [5] Med. Chron., February 1901, p. 366. [6] New York Med. Rec, December 29, 1900, p. 1015. [7] Lancet, January 19, 1901, p. 196; and Boston Med. and Surg. Jour., November 29, 1900. [8] Amer. Jour. of Med. Sciences, December 1900, p. 711. [9] Treatment, November 1900, p. 689. [10] Lancet, October 13, 1900, p. 1061. [11] Clin. Jour., January 13, 1901, p. 214. [12] Annals of Surgery, November 1900, p. 621. [13] Lancet, November 17, 1900.

